# Non-Linear Dynamics in Parkinsonism

**DOI:** 10.3389/fneur.2013.00211

**Published:** 2013-12-25

**Authors:** Olivier Darbin, Elizabeth Adams, Anthony Martino, Leslie Naritoku, Daniel Dees, Dean Naritoku

**Affiliations:** ^1^Department of Neurology, University of South Alabama, Mobile, AL, USA; ^2^Division of System Neurophysiology, National Institute for Physiological Sciences, Okazaki, Japan; ^3^Department of Speech Pathology and Audiology, University of South Alabama, Mobile, AL, USA; ^4^Department of Neurosurgery, University of South Alabama, Mobile, AL, USA

**Keywords:** entropy, EMG, EEG, single unit, movement disorders

## Abstract

Over the last 30 years, the functions (and dysfunctions) of the sensory-motor circuitry have been mostly conceptualized using linear modelizations which have resulted in two main models: the “rate hypothesis” and the “oscillatory hypothesis.” In these two models, the basal ganglia data stream is envisaged as a random temporal combination of independent simple patterns issued from its probability distribution of interval interspikes or its spectrum of frequencies respectively. More recently, non-linear analyses have been introduced in the modelization of motor circuitry activities, and they have provided evidences that complex temporal organizations exist in basal ganglia neuronal activities. Regarding movement disorders, these complex temporal organizations in the basal ganglia data stream differ between conditions (i.e., parkinsonism, dyskinesia, healthy control) and are responsive to treatments (i.e., l-DOPA, deep brain stimulation). A body of evidence has reported that basal ganglia neuronal entropy (a marker for complexity/irregularity in time series) is higher in hypokinetic state. In line with these findings, an entropy-based model has been recently formulated to introduce basal ganglia entropy as a marker for the alteration of motor processing and a factor of motor inhibition. Importantly, non-linear features have also been identified as a marker of condition and/or treatment effects in brain global signals (EEG), muscular activities (EMG), or kinetic of motor symptoms (tremor, gait) of patients with movement disorders. It is therefore warranted that the non-linear dynamics of motor circuitry will contribute to a better understanding of the neuronal dysfunctions underlying the spectrum of parkinsonian motor symptoms including tremor, rigidity, and hypokinesia.

## Introduction

Regarding conditions associated with movement disorders, identification of neuronal correlates to motor symptoms is an important step in characterizing pathological conditions and developing new therapeutics aimed to “correct” or to “normalize” network activity and motoric skills (1, 2). Over the last 30 years, the functions (and dysfunctions) of the sensory-motor circuitry have been mainly conceptualized by linear modelization, which has resulted in two main hypotheses on the occurrence of hypokinesia (i.e., parkinsonism) and hyperkinesia (i.e., dyskinesia). The first hypothesis (e.g., “rate hypothesis”) is based on the circuitry organization of the cortico-basal ganglia-thalamo-cortical loop and its sequential arrangement of excitatory and inhibitory pathways (3, 4). This model postulates that hypokinesia is associated with an imbalanced activity between excitatory and inhibitory drives, in favor of excitation in the output basal ganglia nuclei (e.g., GPi/SNr). The second hypothesis (e.g., “oscillatory hypothesis”) is based on oscillatory activities identified in both single unit and network (i.e., local field potentials, LFPs) activities (5–8). Evidences have linked increased beta band (10–30 Hz) activity to hypokinetic conditions (9–11). In animal models for parkinsonism, the rate and oscillatory hypotheses have generally predicted the effects of anti-parkinsonian treatments on neural activities (i.e., reduced firing rate in GPi/SNr and oscillatory activities in beta band) (4, 12–14), but clinical studies comparing basal ganglia data streams between neurological conditions have reported conflicting data (15–25). In addition to these common models, and based on computational approach, Terman’s group introduced the notion of “connectivity strength” between basal ganglia nuclei as a factor affecting rhythmic firing generation and suggested causal relationship to parkinsonian tremor (26).

The linear analyses used to define basal ganglia activities either in the rate or oscillatory models measure the resultant linear combinations of independent patterns in the data stream. Analyses in the time and frequency domains envisage the interspike interval (ISI) series by the summation of probability distributions for different durations of ISIs (i.e., firing rate, its range or standard deviation) or several frequencies (power spectrum) respectively. These analyses have also been successfully used to characterize the kinetics of movement disorders as well as the EMG in patients with movement disorders and network activities (10, 11).

However, other studies have shown that the irregularity in the neuronal firing activity (27–34), LFPs/EEG (35–38), EMG (39, 40), or kinetics of movement (41, 42) is not random in nature but exhibits a complex temporal organization. In the last decade there has been a growing body of evidence that suggests that linear analyses for basal ganglia circuitry does not fully describe the dynamic nature of these signals, thus justifying the use of non-linear analyses to complement their characterizations (2, 43–47).

In the following sections, we review the findings regarding the non-linear dynamics of physiological functions in Parkinsonism. Finally, we discuss the new challenges and avenues offered by the introduction of these tools to characterize the functions of motor circuitries.

## Non-Linear Systems and Common Tools for Their Investigation Related to Movement Disorders

The term non-linear systems refer to organized systems that generate output activities that are not directly proportional to its input. Non-linear systems exhibit complex dynamics which cannot be fully described by a linear combination of the individual activities of their constituent parts. While, linear analyses mostly focus on central tendencies to describe the status of a system (i.e., mean rate of firing activity, mean power spectrum over period of time), non-linear analyses offer some insights in the organization (if any) of the variability of status of a system by quantifying the persistence of certain patterns or “shift” in the irregularity (or “apparent randomness”) of the time series. Because it isn’t possible to point out a single cause for the complex behaviors in non-linear systems, some analyses may have to account for more than one type of non-linearity and several analytical tools may be required to characterize the behaviors of the system. The fast expansion of the number of non-linear tools makes their systematic review beyond the scope of this article. In the next paragraph, we limit our introduction to non-linear analyses to those most commonly used in the field of motor circuitry activities which is the approximate entropy (ApEn).

As highlighted by Pincus (48), irregularity in a time series results in high standard deviation and unpredictability. While variants of standard deviation appropriately quantify the deviation from centrality (i.e., variability in magnitude), other tools are required to grade the extent of unpredictability (also referred as irregularity or complexity). Irregularity in time series can be graded by exact regularity statistics such as entropy measures. However, their implementations require large set of data free from noise. In real world conditions, and *in vivo* experimentation, these two conditions cannot be respected since recordings are limited to relatively short period of time, and noise originating from the monitoring systems can contaminate the data. Approximate entropy (ApEn) is an approximation of the Kolmogorov–Sinai entropy that was developed by Pincus to provide a tool to grade regularity in short noisy data set. The calculation of ApEn is model-independent; in system biology, this can reduce biases resulting from the assumption on the organization of the data set. ApEn [and its variants SampEn (49)] is a discriminatory tool to distinguish data sets on the basis of regularity. In Pincus’ ApEn, the algorithm uses three parameters to compute the approximation of entropy: (i) the length of the data set (i.e., number of spike intervals), (ii) the embedding dimension (*m*), and (iii) the vector comparison length (*r*). The choice of input parameters has been discussed by Pincus and Goldberger (50) and needs to be in a meaningful range. The authors concluded that, for *m* = 2, values of *r* from 0.1 to 0.25 SD, where SD is the standard deviation of the signal, produce good statistical validity of ApEn and SampEn. Because ApEn value is also dependent on the length of the time series (*N*), the three input parameters must be constant between pairs of compared data sets. A very important property of ApEn is the relative consistency which states that if dataset A is more regular than dataset B for one choice of parameters (*m, r, N*), then it should also exhibit this for all other choices of parameters (49). Finally, by normalizing the vector comparison to the SD of each time series ensuring that ApEn remains unchanged under uniform process magnification (49). In other words, and related to neurophysiological signals, the irregularity of neuronal entropy is decorrelated from the firing rate and the LFP entropy is decorrelated from the amplitude of the signal. Other relatively common tools to investigate the non-linear features in the motor circuitry include the correlation dimension and sample entropy. These values are closely related to the approximate entropy by their algorithms and are reviewed in details elsewhere (51).

## Non-Linear Dynamic of Movement Kinematics and EMG in Parkinsonism

The parkinsonian tremor is a cardinal symptom of the condition which exhibits a modal frequency between 4 and 6 Hz, whereas the postural tremor is between 5 and 12 Hz (52–55). The time-dependent organization of parkinsonian tremor was reported to be more regular (lower approximate entropy, ApEn) in Parkinson’s Disease (PD) patients compared to those with the physiological tremor monitored in the healthy control group (41, 56). Importantly, both STN deep brain stimulation (DBS) and medication reduce tremor regularity (increase entropy) but fail to normalize it to healthy control values (57, 58). Specific to the effects of DBS, tremor entropy decreases with the voltage increase (59). In addition to the tremor, gait kinematics were found less regular in PD patients (higher entropy) than in healthy controls (60–64). As previously observed for the tremor, levodopa partially normalized the regularity of the temporal organization of gait patterns (65).

Differences in EMGs characteristics between PD patients and healthy persons include an increased tonic background activity (66), increased synchronization in 8–12 Hz, decreased amplitude in the 20–25 Hz (56, 67), and an alternating pattern of EMG bursts during voluntary movement (68). In the non-linear domain, both EMG and acceleration signals exhibit lower complexity (lower entropy) in PD patients than in healthy subjects (56). The complexity of the PD tremor was found to be further reduced by anti-parkinsonian treatments including DBS (57, 69).

The identification of non-linear features in the kinetics of some disorders and their related EMG activities has therefore raised the question of whether there could be central changes in non-linear features of sensory-motor circuitry activities.

## Non-Linear Dynamic in Network and Neuronal Datastream in Parkinsonism

Stam et al. (70, 71) identified non-linear features in EEGs recorded from Parkinsonian patients. Later, entropy (or complexity) of EEGs was reported to be increased in PD patients comparatively to healthy controls (72). This finding was confirmed by Han et al. who further indicated that PD is associated with an increased complexity of the EEG’s rhythm (73).

Non-linear temporal organization has also been identified in the interval interspikes series (ISIs) recorded from basal ganglia neurons in the awake normal primate and in Parkinsonian patients (47). These analyses have established that the non-linear temporal organization of ISIs in the time series results in the replication of complex patterns that cannot be statistically explained by random trials from the probability distribution of the ISIs (2, 47). In other words, the non-linear temporal organization of basal ganglia ISIs is less irregular than what its probability distribution would suggest if ISIs were randomly sorted. Yet, the clinical relevance of these neuronal non-linear features to movement disorders is not clear. A retrospective analyses of a database of PD and dystonia neurons with temporal organizations [as defined in Ref. (2)], Sanghera et al. (74) found a lower neuronal entropy in the GPi of dystonia patients comparatively to PD patients. The evidence that changes in non-linear features may account for the effects of treatments for movement disorders was initially established by Dorval and colleagues (43). This study in the Parkinsonian primate reported a change in non-linear features in GPi neuronal data stream following DBS of the STN, a treatment effective both for Parkinson Disease and dystonia (75). We have confirmed this finding in Parkinsonian patients by establishing that the dopaminergic agonist apomorphine, administered during DBS operative procedure, decreases neuronal entropy in the STN (46). This finding has also helped establish a first link between neurotransmission (especially dopaminergic) and neuronal entropy.

**Figure 1:**
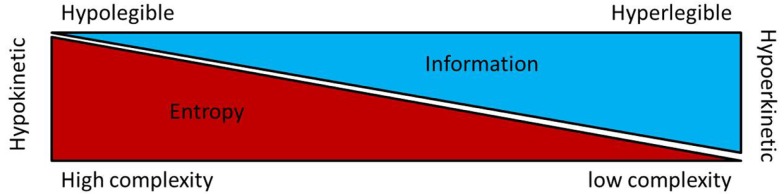
**This figure shows a putative relationship between entropy, information and kinesia regarding the motoric functions of the basal ganglia**. In the so called “Entropy hypothesis,” increase entropy (or irregularity) in the firing rate of STN and/or GPi neurons result in loss of information [Shannon–Brillouin’s interpretation of entropy (76)] and decreased kinesia [for details, see Ref. (80)].

Taken together, the available data on non-linear dysfunctions of sensory-motor circuit have suggested that the hypokinetic condition is associated to higher entropy at least in the GPi and STN. Recently, the basal ganglia neuronal entropy was introduced as a putative interfering factor in the current model for the selection and the inhibition of motor program. The entropy-based model for basal ganglia dysfunctions in movement disorders envisage neuronal entropy under Shannon–Brillouin interpretation (76) as a measure of disorder, unpredictability and reduced motor information. Under this hypothesis high neuronal entropy in the STN and GPi neuronal data stream is interpreted as a network condition generating a large number different pattern possibilities leading to a signal with limited order or “organization” and reduced information. In regard to the concept of selection and inhibition of motor program along the basal ganglia circuitry (77), the entropy-hypothesis introduces complexity of neuronal data stream as a factor which enhances the inhibition of motor program by decreasing its informative nature (see Figure 1). In the parkinsonian state, the “entropy hypothesis” predicts that high STN and GPi neuronal entropies would decrease information in the data stream and motor program selection resulting in hypokinesia. There is currently no clinical or experimental data available to directly relate the changes in BG neuronal entropy to circuitry alterations resulting in the parkinsonian states. In theory, the entropy hypothesis suggests that high GPi neuronal entropy underlies an inadequate compression [or reduction of the dimensionality (78)] of up-stream population activity into the output neurons of the BG circuitry (78). This could be the consequence of the striatal dopaminergic depletion (46) and/or some circuitry reorganizations such as increased interconnections between elements of the motor circuitry (79). Experimental research in animal models for movement disorders is warranted to explore these avenues.

## Discussion

Investigation of non-linear dynamics associated to parkinsonism is still in its infancy but available data shows that both conditions and treatments affect the complex temporal organizations found in motoric kinetics, EMG, global signals, and basal ganglia neuronal activities.

Today, the drawing of a non-linear functional model related to the anatomo-pathology of the parkinsonian basal ganglia is not reachable yet. Though it is remarkable that both global signals (EEG) and single unit activities show entropy increases in parkinsonism, the lack of well-controlled comparisons between pathological and normal states of motor-related territories remains an issue in interpreting these data in regard to the effects of the conditions *per se* on movement disorders. In addition, the lack of data on the relationships between neuronal entropy and global signal entropy, as well as on the inter-nuclei entropy relationships (2, 47, 81) continue to be major obstacles to linking the alteration of non-linear basal ganglia dynamic to the histological changes of the circuitry associated to the PD condition. A systemic evaluation of the main neurotransmitters on the non-linear dynamic of basal ganglia neurons is necessary to draw a non-linear functional-anatomical model of the motor circuitry both in the normal and the pathological conditions. Single unit recordings combined with LFPs in an animal model for movement disorders (82, 83), and in patients (84), will certainly help to progress in this research direction.

The use of non-linear domain analyses to describe the changes in motor-related dynamics is warranted to provide new qualitative and quantitative information regarding the nature of the alterations in sensory-motor processing associated to the parkinsonian condition. Entropy is a general and commonly used parameter in this new field. The fact that this feature can be followed from the neuronal activity up to the kinetics of movement (via the EEG and EMG) offers an interesting opportunity to investigate the motor information downstream from the central level up to the effectors under a single framework but without warranting a causal relationship. Arguably, this may be less directly accessible within the framework of the rate or oscillatory hypotheses. However, the aim of entropy-related algorithms (i.e., ApEn/SampEn) is not to identify the complex patterns in the time series. Additional non-linear analytical tools need to be integrated in order to better identify non-linear hallmarks in the neuronal and global signals. The identification of such patterns could become relevant in future algorithms for closed-loop devices aimed to deliver on-demand anti-parkinsonian treatments (85). An additional research orientation would be to investigate whether the delivery of non-linear complex patterns could improve the benefit of DBS.

## Conclusion

The use of non-linear domain analyses to describe the neuronal and network activities inside the basal ganglia may provide new qualitative and quantitative information relative to the nature of the sensory-motor processing, as well as its distortion in pathological conditions. It is expected that the inclusion of key non-linear features into silicone-based models of the basal ganglia could better reproduce the complexity and non-stationarity of signals recorded in normal and pathological conditions. In translational research, non-linear analytical tools may provide new strategies to improve the efficiency of brain-interfaces and other closed-loop systems aimed to control therapeutic delivery in movement disorders.

## Conflict of Interest Statement

The authors declare that the research was conducted in the absence of any commercial or financial relationships that could be construed as a potential conflict of interest.
